# Sixth Annual Report of the Ohio Lunatic Asylum

**Published:** 1845-02

**Authors:** 


### SIXTH ANNUAL REPORT OF THE OHIO LUNATIC ASYLUM.

In acknowledging the receipt of this Report, we are happy to say, that it presents the Asylum as everyway prosperous and successful. In six years, 541 patients have been admitted, of whom 243 have been cured, and 23 discharged in an improved condition; making together but a fraction less than half. Of this number 195 were recent cases, and 71 old, or of more than a year's duration, before admission. These two facts are worthy of all attention. The first shows, that insanity is by no means an incurable malady; the second, that its curability is in proportion to the early stage of the disease, in which treatment is commenced. In private practice, such treatment, that is the appropriate, is seldom practicable; and we know of no disease, which more imperatively requires to be made a *speciality* than this. The number of cases which ordinarily fall to the lot of a private practitioner, are too few to give him the requisite experience; and, with very few exceptions, whatever, may be his skill, his prescriptions, both medicinal and moral, cannot be carried out, while the patient remains at home. Every State in the Union ought to have one or more hospitals for the insane. In the Valley of the Mississippi, Kentucky, Ohio, Tennessee, and Louisiana have erected them; and Indiana has moved in the good work; but, we believe, Alabama, Mississippi, Arkansas, Missouri, and Illinois, have not as yet entered upon it. We hope our brethren in those States will do justice to our benevolent profession, by reminding their legislators of their duty in this matter.

In casting our eyes over that part of the Report which was contributed by the indefatigable medical superintendant of the establishment, Dr. Awl, we find the following fact, which seems worthy of being placed before the profession:

"The months of December, January, and February, 1843–'4, were particularly severe upon our epileptic patients. Their convulsive paroxysms were increased in number and severity, followed by an extraordinary sinking and prostration of the vital powers, occasioning death in the three instances mentioned, and an unusual amount of suffering and weakness of body and mind, for many days, in all who survived. We observed something of the same kind of suffering with this class of patients, about two years since, when, as upon the last occasion, it appeared to assume the form of a special endemic amongst the epileptics. All others continued to enjoy their ordinary health. It was characterized by very frequent returns of the epileptic spasms, with strong tendency to apoplexy. The pulse was remarkably small, weak, and fluttering; features dull, unmeaning, and sunken; mind lost, or imbecile, attended with great exhaustion of physical strength. On both occasions, it was wonderful to observe how the most robust were enfeebled and fallen, and how long time it required for them to regain their former strength and condition of mind and body. A hired girl in the Institution, subject to occasional epilepsy, was also observed to suffer from the same train of symptoms. With the last appearance of this peculiar endemic, the weather was unusually mild and open for the season, and the country extensively inundated, from continued rains; the thermometer being greatly elevated, and the barometer depressed, for a long period of time."

For the information of such of our readers, as, residing in States which have no asylums, might direct their insane patients to be taken to that of Ohio, we may as well state, that they cannot be received, as the present buildings are not sufficient for the insane of that State, and new ones are now in the course of erection. Even the present edifice, however, is the largest in the Valley of the Mississippi, and the institution, in all respects, must be admitted to stand before every other in the West. It may, indeed, be advantageously compared with the best eastern institutions, for while it is inferior to some, it rises above many others, in the number of its patients, and the success attendant on their treatment.

